# Taxonomy and phylogenetic appraisal of *Spegazzinia musae* sp. nov. and *S. deightonii* (Didymosphaeriaceae, Pleosporales) on Musaceae from Thailand

**DOI:** 10.3897/mycokeys.70.52043

**Published:** 2020-07-21

**Authors:** Binu C. Samarakoon, Rungtiwa Phookamsak, Dhanushka N. Wanasinghe, Putarak Chomnunti, Kevin D. Hyde, Eric H. C. McKenzie, Itthayakorn Promputtha, Jian-Chu Xu, Yun-Ju Li

**Affiliations:** 1 The State Phosphorus Resource Development and Utilization Engineering Technology Research Centre, Yunnan Phosphate Chemical Group Co. Ltd, Kunming 650201, China; 2 School of Science, Mae Fah Luang University, Chiang Rai 57100, Thailand; 3 Center of Excellence in Fungal Research, Mae Fah Luang University, Chiang Rai 57100, Thailand; 4 Key Laboratory for Plant Biodiversity and Biogeography of East Asia (KLPB), Kunming Institute of Botany, Chinese Academy of Sciences, Kunming 650201, Yunnan, China; 5 East and Central Asia Regional Office, World Agroforestry Centre (ICRAF), Kunming 650201, Yunnan, China; 6 Honghe Center for Mountain Futures, Kunming Institute of Botany, Chinese Academy of Sciences, Honghe County, Yunnan, China; 7 Institute of Plant Health, Zhongkai University of Agriculture and Engineering, Guang Dong Province, China; 8 Landcare Research Manaaki Whenua, Private Bag 92170, Auckland, New Zealand; 9 Department of Biology, Faculty of Science, Chiang Mai University, Chiang Mai 50200, Thailand; 10 Research Center in Bioresources for Agriculture, Industry and Medicine, Chiang Mai University, Chiang Mai 50200, Thailand

**Keywords:** Ascomycota, Dothideomycetes, fungi on banana, Hyphomycetes, Thai mycobiota

## Abstract

Tropical plants host a range of fungal niches including endophytes, pathogens, epiphytes and saprobes. A study undertaken to discover the saprobic fungal species associated with *Musa* sp. (banana) from northern Thailand found two hyphomycetous taxa of *Spegazzinia* (Didymosphaeriaceae, Pleosporales). These were collected during the dry season and their morpho-molecular taxonomic relationships were investigated. Based on phylogenetic analysis of combined SSU, LSU, ITS and TEF1-α sequence data (77% ML, 0.99 BYPP) and contrasting morphological features to the sister taxon, we introduce *Spegazzinia musae* as a novel species from a decaying leaf of *Musa* sp. Details on the taxonomy, ecology and geographical distribution of *Spegazzinia* species are provided. In addition, we report *S. deightonii* as a new host record from *Musa* sp. Our data further validate the taxonomic placement of *Spegazzinia* in Didymosphaeriaceae.

## Introduction

Several taxonomic studies have been conducted to assess the saprobic fungal diversity in *Musa* species (Ellis 1971, 1976; Matsushima 1971; Photita et al. 2001b; Somrithipol 2007; Hernández-Restrepo et al. 2015; Crous et al. 2016; Hyde et al. 2017). Ellis (1971) described several species on *Musa* (i.e. *Arthrinium sacchari*, *Cladosporium musae*, *Cordana musae*, *Curvularia fallax*, *Deightoniella torulosa*, *Gliomastix elata*, G. murorum var. polychroma, *G. musicola*, *Gyrothrix hughesii*, *Haplobasidion musae*, *Memnoniella subsimplex*, *Periconia digitata*, *P. lateralis*, *Periconiella musae*, *Pithomyces sacchari*, *Pyriculariopsis parasitica*, *Spegazzinia tessarthra*, *Stachylidium bicolor*, *Tetraploa aristata*, *Zygosporium gibbum*, *Z. masonii* and *Z. minus*). Ellis (1976) also described *Bidenticula cannae*, *Chlamydomyces palmarum*, *Cordana johnstonii*, *Parapyricularia musae* and *Veronaea musae* on *Musa* sp. Photita et al. (2001b) identified 46 saprobic fungal taxa from *Musa acuminata* in Hong Kong. Most of the saprobes reported by Photita et al. (2001b) belonged to the genera *Anthostomella*, *Deightoniella*, *Durispora*, *Hansfordia*, *Memnoniella*, *Nigrospora*, *Pyriculariopsis*, *Pseudopithomyces*, *Verticillium* and *Zygosporium*. In addition, *Dictyoarthrinium* (Somrithipol 2007) and *Ramichloridium* (Kirschner and Piepenbring 2014) were also recorded as saprobes on *Musa* sp. Considering the economic importance of *Musa* sp. there are not many studies on the saprobic fungal populations associated with this host. Few studies have molecular data for the identified strains. To address this research gap, we are investigating the saprobic fungal diversity of *Musa* sp. in the Asian region where the fungi are highly diverse (Hyde et al. 2018).

*Spegazzinia* was established by Saccardo (1880) based on *S. ornata*. Currently 17 taxa are listed in Species Fungorum (2020). Based on morphology, the genus was placed in Apiosporaceae (Sordariomycetes) by Hyde et al. (1998). Based on SSU, LSU, ITS and TEF1-α sequence data of *S. deightonii* and *S. tessarthra*, Tanaka et al. (2015) placed *Spegazzinia* in Didymosphaeriaceae (Dothideomycetes). This was supported by a phylogenetic analysis which placed *Spegazzinia* in a basal clade in Didymosphaeriaceae (Thambugala et al. 2017).

Hughes (1953) characterized *Spegazzinia* as a hypomycetous taxon with a unique basauxic conidiophore ontogeny (conidiophores that arise and elongate from a cup-shaped basal cell called a conidiophore mother cell). The conidia of *Spegazzinia* are brown to dark brown and dimorphic in most species, with a disc-shaped form and a stellate form (Ellis 1971; Manoharachary and Kunwar 2010). However, little molecular data for this genus is available in the GenBank (https://www.ncbi.nlm.nih.gov/). Therefore, for a better phylogenetic resolution of the genus in Didymosphaeriaceae, the previously identified taxa should be recollected to obtain DNA sequence data and morphological descriptions.

In this present study, we introduce *Spegazzinia musae* sp. nov. and report the first occurrence of *Spegazzinia deightonii* from *Musa* sp. in Thailand. We provide detailed morphological descriptions, illustrations and molecular justification for the introduction of *Spegazzinia musae* sp. nov. Our molecular analyses further support the phylogenetic placement of *Spegazzinia* in Didymosphaeriaceae.

## Materials and methods

### Sample collection, morphological studies and isolation

Dead plant materials of *Musa* sp. (banana) were collected from Thailand during the dry season of 2018 to 2019. Specimens were transferred to the laboratory in cardboard boxes. Samples were examined with a Motic SMZ 168 Series microscope. Powdery masses of conidia were mounted in water for microscopic studies and photomicrography. The taxa were examined using a Nikon ECLIPSE 80i compound microscope and photographed with a Canon 550D digital camera fitted to the microscope. Measurements were made with the Tarosoft (R) Image Frame Work program and images used for figures processed with Adobe Photoshop CS3 Extended version 10.0 software (Adobe Systems, USA).

Single spore isolation was carried out following the method described in Chomnunti et al. (2014). Germinated spores were individually transferred to potato dextrose agar (PDA) plates and grown at 25 °C in daylight. Colony characteristics were observed and measured after 3 weeks. Specimens were deposited in the Mae Fah Luang University (**MFLU**) Herbarium, Chiang Rai, Thailand. Living cultures were deposited in the Culture Collection of Mae Fah Luang University (**MFLUCC**).

### DNA extraction and PCR amplification

Fungal isolates were grown on PDA for 4 weeks at 25 °C and total genomic DNA was extracted from 50 to 100 mg of axenic mycelium of the growing cultures according to Wanasinghe et al. (2018). The mycelium was ground to a fine powder with liquid nitrogen and fungal DNA was extracted using the Biospin Fungus Genomic DNA Extraction Kit-BSC14S1 (BioFlux, P.R. China) according to the instructions of the manufacturer. Four gene regions, the internal transcribed spacer (ITS), partial 18S small subunit (SSU), partial 28S large subunit (LSU), and partial translation elongation factor 1-alpha gene (TEF1-α) were amplified using ITS5/ITS4 (White et al. 1990), NS1/NS4 (White et al. 1990), LR0R/LR5 (Vilgalys and Hester 1990) and EF1-983F/EF1-2218R (Rehner 2001) primers, respectively.

Polymerase chain reaction (PCR) was conducted according to the following protocol. The total volume of the PCR reaction was 25 μL containing 12.5 μL of 2 × Power Taq PCR MasterMix (a premix and ready to use solution, including 0.1 Units/ μL Taq DNA Polymerase, 500 μm dNTP Mixture each (dATP, dCTP, dGTP, dTTP), 20 mM Tris-HCL pH 8.3, 100 mM KCl, 3 mM MgCl_2_, stabilizer and enhancer), 1 μL of each primer (10 pM), 2 μL genomic DNA template and 8.5 μL double distilled water (ddH_2_O). The reaction was conducted by running for 40 cycles. The annealing temperature was 56 °C for ITS and LSU, 57.2 °C for TEF1-α and 55 °C for SSU and initially 95 °C for 3 mins, denaturation at 95 °C for 30 seconds, annealing for 1 min, elongation at 72 °C for 30 seconds, and final extension at 72 °C for 10 mins for all gene regions. PCR amplification was confirmed on 1% agarose electrophoresis gels stained with ethidium bromide. The amplified PCR fragments were sent to a commercial sequencing provider (TsingKe Biological Technology (Beijing) Co., Ltd, China). The nucleotide sequence data acquired were deposited in GenBank.

### Sequencing and sequence alignment

Obtained sequences were subjected to BLASTn search in GenBank (https://blast.ncbi.nlm.nih.gov/Blast.cgi). BLASTn search results and initial morphological studies supported that our isolates belonged to Didymosphaeriaceae. Other sequences used in the analyses were obtained from GenBank based on recently published data (Tanaka et al. 2015; Jayasiri et al. 2019) (Table 1). The single gene alignments were automatically done by MAFFT v. 7.036 (http://mafft.cbrc.jp/alignment/server/index.html, Katoh et al. 2019) using the default settings and later refined where necessary, using BioEdit v. 7.0.5.2 (Hall 1999). The finalized alignment and tree were submitted to TreeBASE (submission ID: 25686, http://www.treebase.org/).

**Table 1.** Taxa used in the phylogenetic analysis of *Spegazzinia* with the corresponding GenBank accession numbers. Type strains are superscripted with T and newly generated strains are indicated in bold.

Species	Strains *	GenBank accession numbers				References
		LSU	SSU	ITS	TEF1-α	
*Alloconiothyrium aptrootii*	CBS 980.95^T^	JX496234	–	JX496121	–	Verkley et al. (2014)
*Bimuria novae zelandiae*	CBS 107.79^T^	AY016356	AY016338	–	–	Lumbsch and Lindemuth (2001)
*Dendrothyrium variisporum*	CBS 121517^T^	JX496143	–	JX496030	–	Vu et al. (2019)
*Deniquelata barringtoniae*	MFLUCC 110422^T^	JX254655	JX254656	JX254654	–	Ariyawansa et al. (2013)
*Didymocrea sadasivanii*	CBS 438.65^T^	DQ384103	DQ384066	–	–	Vu et al. (2019)
*Didymosphaeria rubi ulmifolii*	MFLUCC 14-0023^T^	KJ436586	KJ436588	–	–	Ariyawansa et al. (2014)
*Kalmusia spartii*	MFLUCC 14-0560^T^	KP744487	KP753953	KP744441	–	Liu et al. (2015)
*Karstenula rhodostoma*	CBS 690.94	GU301821	GU296154	–	–	Schoch et al. (2009)
*Laburnicola muriformis*	MFLUCC 16-0290^T^	KU743198	KU743199	KU743197	KU743213	Wanasinghe et al. (2016)
*Montagnula cirsii*	MFLUCC 13-0680^T^	KX274249	KX274255	KX274242	KX284707	Hyde et al. (2016)
*Montagnula graminicola*	MFLUCC 13-0352^T^	KM658315	KM658316	KM658314	–	Liu et al. (2015)
*Neokalmusia brevispora*	KT 2313^T^	AB524601	AB524460	–	AB539113	Tanaka et al. (2009)
*Neokalmusia scabrispora*	KT 2202	AB524594	AB524453	–	AB539107	Tanaka et al. (2009)
*Paracamarosporium hawaiiense*	CBS 120025^T^	JX496140	EU295655	JX496027	–	Verkley et al. (2014)
*Paraconiothyrium cyclothyrioides*	CBS 972.95^T^	JX496232	AY642524	JX496119	–	Verkley et al. (2014)
*Paraconiothyrium estuarinum*	CBS 109850^T^	JX496129	AY642522	JX496016	–	Verkley et al. (2014)
*Paramassariosphaeria clematidicola*	MFLU 16-0172^T^	KU743207	KU743208	KU743206	–	Wanasinghe et al. (2016)
*Paraphaeosphaeria michotii*	MFLUCC 13-0349^T^	KJ939282	KJ939285	KJ939279	–	Tennakoon et al. (2016)
*Phaeodothis winteri*	AFTOL-ID 1590	DQ678073	DQ678021	–	DQ677917	Schoch et al. (2006)
*Pleospora herbarum*	CBS 191.86^T^	GU238160	GU238232	–	KC584731	Aveskamp et al. (2010)
*Pseudocamarosporium cotinae*	MFLUCC 14-0624^T^	KP744505	KP753964	KP744460	–	Liu et al. (2015)
*Pseudocamarosporium propinquum*	MFLUCC 13-0544^T^	KJ813280	KJ819949	KJ747049	–	Wijayawardene et al. (2014)
*Pseudopithomyces chartarum*	UTHSC 04-678	HG518065	–	HG518060	–	Da Cunha et al. (2014)
*Spegazzinia bromeliacearum*	URM 8084^T^	MK809513	–	MK804501	–	Crous et al. (2019)
*Spegazzinia deightonii*	yone 212	AB807582	AB797292	–	AB808558	Tanaka et al. (2015)
***Spegazzinia deightonii***	**MFLUCC 20-0002**	**MN956772**	**MN956770**	**MN956768**	**MN927133**	This study
*Spegazzinia deightonii*	yone 66	AB807581	AB797291	–	AB808557	Tanaka et al. (2015)
*Spegazzinia intermedia*	CBS 249.89	MH873861	–	MH862171	–	Vu et al. (2019)
*Spegazzinia lobulata*	CBS 361.58	MH869344	–	MH857812	–	Vu et al. (2019)
***Spegazzinia musae***	**MFLUCC 20-0001^T^**	**MN930514**	**MN930513**	**MN930512**	**MN927132**	This study
*Spegazzinia neosundara*	MFLUCC 15–0456^T^	KX954397	KX986341	KX965728	–	Thambugala et al. (2017)
*Spegazzinia radermacherae*	MFLUCC 17-2285^T^	NG_066308	MK347848	NR_163331	MK360088	Jayasiri et al. (2019)
*Spegazzinia* sp.	yone 279	AB807583	AB797293	–	AB808559	Tanaka et al. (2015)
*Spegazzinia tessarthra*	SH 287	AB807584	AB797294	–	AB808560	Tanaka et al. (2015)
*Stemphylium botryosum*	CBS 714.68^T^	KC584345	KC584603	KC584238	KC584729	Woudenberg et al. (2013)
*Tremateia arundicola*	MFLU 16-1275^T^	KX274248	KX274254	KX274241	KX284706	Tennakoon et al. (2016)
*Tremateia guiyangensis*	GZAAS01^T^	KX274247	KX274253	KX274240	KX284705	Tennakoon et al. (2016)
*Xenocamarosporium acaciae*	CPC 24755^T^	KR476759	–	KR476724	–	Tennakoon et al. (2016)

*Abbreviations of culture collections: **AFTOL-ID**: Assembling the Fungal Tree of Life, **CBS**: Westerdijk Fungal Biodiversity Institute, Utrecht, Netherlands, **CPC**: Working collection of Pedro Crous housed at CBS, **GZAAS**: Guizhou Academy of Agricultural Sciences herbarium, China, **KT**: K. Tanaka, **MFLU**: Mae Fah Luang University, Chiang Rai, Thailand, **MFLUCC**: Mae Fah Luang University Culture Collection, Chiang Rai, Thailand, **SH**: Academia Sinica People’s Republic of China. Shanghai, **URM**: Universidade Federal de Pernambuco, **UTHSC**: Fungus Testing Laboratory, University of Texas Health Science Center, San Antonio, Texas, USA, **Yone**: H. Yonezawa.

### Phylogenetic analysis

**Figure 1.**
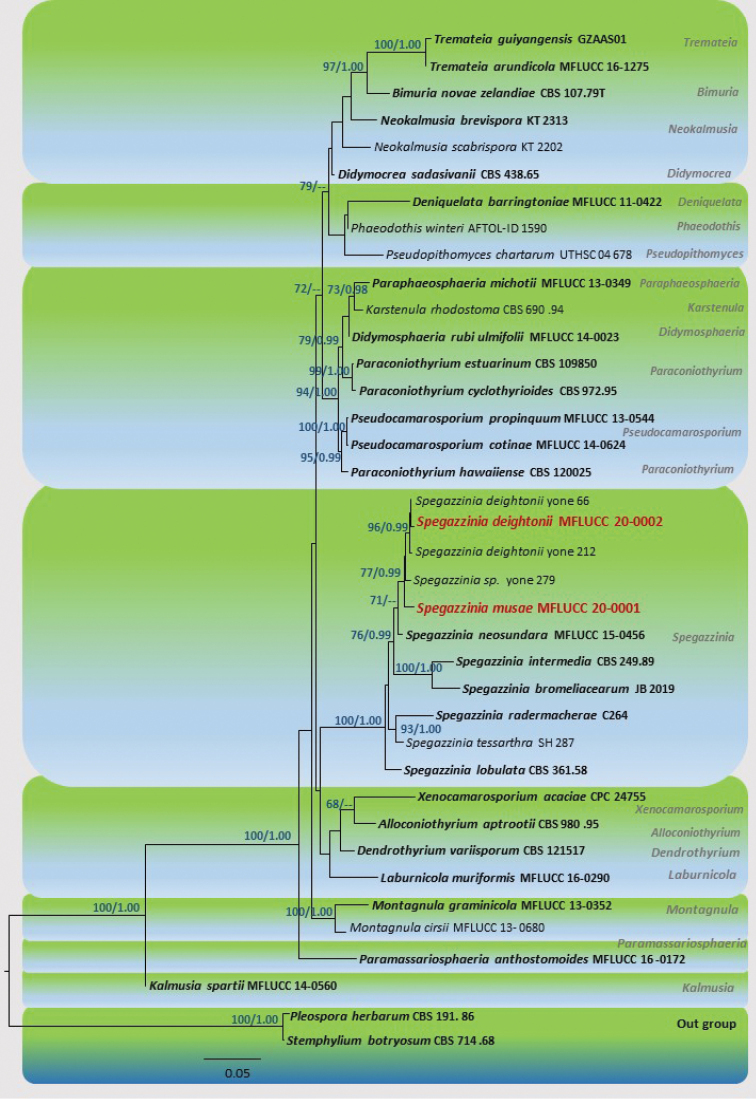
Maximum likelihood tree revealed by RAxML from an analysis of SSU, LSU and ITS and TEF1-α sequence data of selected genera of family Didymosphaeriaceae, showing the phylogenetic position of *Spegazzinia musae* (MFLUCC 20-0001) and *S. deightonii* (MFLUCC 20-0002). ML bootstrap supports (≥60 %) and Bayesian posterior probabilities (≥ 0.95 BYPP) are given above in the branches, respectively. The tree was rooted with *Pleospora herbarum* and *Stemphylium botryosum* (Pleosporaceae). Strains generated in this study are indicated in red-bold. Ex-type species are indicated in bold. The scale bar represents the expected number of nucleotide substitutions per site. A best scoring RAxML tree is shown with a final ML optimization likelihood value of -13516.66. The matrix had 795 distinct alignment patterns, with 33.60% of undetermined characters or gaps. Estimated base frequencies were: A = 0.239862, C = 0.245185, G = 0.277025, T = 0.237927; substitution rates AC = 1.626982, AG = 2.468452, AT = 1.211822, CG = 1.092437, CT = 6.295657, GT = 1.000000; proportion of invariable sites I = 0.484119; gamma distribution shape parameter α = 0.445929.

Maximum likelihood (ML) trees were generated using the RAxML-HPC2 on XSEDE (8.2.8) (Stamatakis et al. 2008; Stamatakis 2014) in the CIPRES Science Gateway platform (Miller et al. 2010) using GTR+I+G model of evolution. Bootstrap support was obtained by running 1000 pseudo-replicates. Maximum likelihood bootstrap values (ML) equal or greater than 60% are given above each node in blue (Figure 1).

A Bayesian inference analysis was conducted with MrBayes v. 3.1.2 (Huelsenbeck and Ronquist 2001) to evaluate posterior probabilities (PP) (Rannala and Yang 1996; Zhaxybayeva and Gogarten 2002) by Markov chain Monte Carlo sampling (BMCMC). Two parallel runs were conducted, using the default settings, but with the following adjustments: four simultaneous Markov chains were run for 2,000,000 generations and trees were sampled every 100^th^ generation and 20,000 trees were obtained. The first 4,000 trees, representing the burning phase of the analyses were discarded. The remaining 16,000 trees were used for calculating PP in the majority rule consensus tree. Branches with Bayesian posterior probabilities (BYPP) greater than 0.95 are indicated above each node in blue (Figure 1). Phylograms were visualized with FigTree v1.4.0 program (Rambaut 2011) and reorganized in Microsoft Power Point.

### Data resources

The data underpinning the analysis reported in this paper are deposited in the Dryad Data Repository at https://doi.org/10.5061/dryad.2ngf1vhk6.

## Results

### Phylogenetic analysis

The combined SSU, LSU, ITS, TEF1-α matrix comprised 38 sequences including selected genera in Didymosphaeriaceae. A best scoring RAxML tree is shown in Figure 1. All trees (ML and BYPP) were similar in topology and did not differ (data not shown) at the generic relationships which are in agreement with multi-gene phylogeny of Tanaka et al. (2015). All *Spegazzinia* strains analyzed here were clustered as a highly supported monophyletic clade (100% ML, 1.00 BYPP) in Didymosphaeriaceae (Figure 1) sister to *Alloconiothyrium*, *Dendrothyrium*, *Laburnicola* and *Xenocamarosporium*. Our new species, *Spegazzinia musae* (MFLUCC 20-0001) clustered with *Spegazzinia* sp. (yone 279) and *S. deightonii* (yone 66, MFLUCC 20-0002, yone 212) with significant statistical support (77% ML, 0.99 BYPP). Strain MFLUCC 20-0002 grouped with *S. deightonii* (yone 66, yone 212) with high statistical support (96% ML, 0.99 BYPP).

### Taxonomy

#### Spegazzinia deightonii

Taxon classificationFungiPleosporalesDidymosphaeriaceae

(S. Hughes) Subram., J. Indian bot. Soc. 35: 78 (1956)

C429487B-B639-5CF2-A5B5-E45B14E51180

Facesoffungi Number: FoF07238

Figure 2

##### Description.

*Saprobic* on dead leaves of *Musa* sp. **Sexual morph** Undetermined. **Asexual morph** Hyphomycetous. *Sporodochia* powder like, dark, dense, dry, 1–3 mm diameter. *Conidiophore mother cells* 3.5–6.8 × 2.5–5.0 μm (*x̄* = 5.59 × 4.15 μm, n = 6), hyaline to light brown, subspherical or doliiform. *Conidiophores* long or short and give rise to two types of conidia referred here as α and β. *Conidiophores of α conidia* up to 48–120 × 1–2 μm (*x̄* = 95.3 × 1.6 μm, n = 20) long, erect or flexuous, narrow, verruculose, unbranched, hyaline to golden-brown. *Conidiophores of β conidia* initially hyaline, light brown to brown at maturity, very short and slightly bent, 1.6–2 × 2.5–3 μm (*x̄* = 1.8 × 2.6 μm, n =10). *Conidiogenous cell development* basauxic, forming a single, terminal holoblastic conidium at the apex of conidiophore. *Conidial development* holoblastic. *Conidia* two types: α *conidia* stellate, 18–28 × 17–29 μm (*x̄* = 25.1 × 23.3 μm, n = 25), solitary, globose to variously shaped, with spines 4–6 μm long, 4–8-celled, frequently 4- to 6-celled, deeply constricted at the septa. β *conidia* disc-shaped, initially hyaline, light brown to dark brown at maturity, 8-celled, 16–21 × 11–14 μm (*x̄* = 19.2 × 14.6 μm, n = 25), flat from both sides with short and blunt spines, frequently with attached conidiogenous cells when splitting from the conidiophores.

##### Culture characteristics.

Conidia germinating on PDA within 13–14 h. Colonies growing on PDA, reaching a diameter of 55 mm after 14 d at 25 °C, raised, moderately dense, undulate margin, middle grey, periphery brownish grey and olive green at immature stage; reverse white to greyish white.

##### Material examined.

Thailand, Chiang Rai Province, Doi Thun, on a dead leaf of *Musa* sp. (Musaceae), 7 December 2018, M.C. Samarakoon, BNS 072 (MFLU 19-2908), living culture MFLUCC 20-0002.

##### Notes.

*Spegazzinia deightonii*MFLUCC 20-0002 clustered with *S. deightonii* (yone 66, yone 212) with significant statistical support (Figure 1). All the strains of *S. deightonii* described in Ellis (1971) and Tanaka et al. (2015) have similar morphological features with our strain such as dark brown, 8-celled, disked-shaped, spiny conidia. With morphological and multigene phylogenetic support, we report a new host record of *S. deightonii* from *Musa* sp.

**Figure 2.**
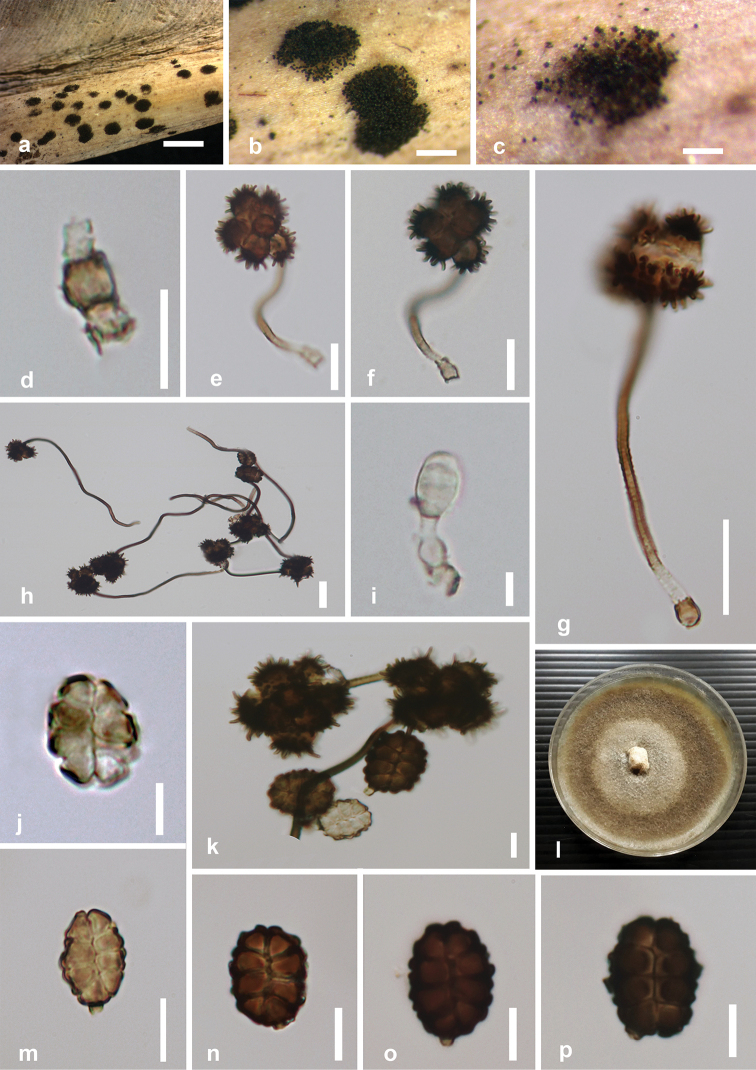
*Spegazzinia deightonii* (MFLU 19-2908) **a–c** fungal colonies on host surface **d** conidiophore mother cell of α conidia **e–g** α conidia **i** a developmental stage of β conidia **h, k** conidia **l** colonies on PDA after 28 days showing sporulation **j, m–p** β conidia. Scale bars: 500μm (**a**), 200μm (**b**), 50 μm (**c**), 20μm (**e–h**), 10μm (**d, k, m–p**), 5 μm (**i, j**).

#### Spegazzinia musae

Taxon classificationFungiPleosporalesDidymosphaeriaceae

Samarakoon, Phookamsak, Wanas., Chomnunti & K.D. Hyde sp. nov.

888C2C14-2FA0-5BEE-878F-04509EB0C4E1

835298

Facesoffungi Number: FoF07237

Figure 3

##### Etymology.

The name reflects the host genus, *Musa* (Musaceae).

##### Holotype.

MFLU 19-2907

##### Description.

*Saprobic* on a dead leaf of *Musa* sp. **Sexual morph** Undetermined. **Asexual morph** Hyphomycetous. *Sporodochia* dark, dense, dry, powdery, velvety, 1–2 mm diameter. *Conidiophore mother cells* 3.4–5.8 × 3.7–4.7 μm (*x̄* = 4.6 × 4.1 μm, n = 10) subhyaline or light brown, doliiform or subspherical. *Conidiophores* usually short to long bearing two types of conidia referred to here as α and β. *Conidiophores* of α *conidia* up to 40–85 × 0.8–2.5 μm (*x̄* = 64 × 21.7 μm, n = 15), pale brown or dark golden brown, rough-walled, hyaline at bottom near the conidiophore mother cell, pale brown at middle, dark golden brown at top near conidial cells, erect or flexuous, narrow and long, generally unbranched, rarely branched. *Conidiophores* of β *conidia* 0.7–3.5 × 1.5–3 μm (*x̄* = 1.9 × 2.3 μm, n = 15) short, erect, unbranched, hyaline when immature, subhyaline or hyaline at maturity. *Conidiogenous cell development* basauxic, forming a single, terminal holoblastic conidium at the apex of conidiophore. *Conidial development* holoblastic. *Conidia* solitary, dry, two types: α *conidia* stellate, 15–22.7 × 14.5–20.5 μm (*x̄* = 18.8 × 17.8 μm, n = 15), 4–6 celled, each cell globose to subglobose, deeply constricted at the septa, conspicuously spinulate, 4–6 spines, each 2–8 μm long arise from surface of each cell. β *conidia* disc-shaped, initially hyaline, 4-celled, each cell slightly turbinate in shape, rough-walled, crossed septate, becoming brown to dark brown at maturity, each cell turbinate, crossed-septate, smooth-walled, light brown at the center near the septa, dark brown at periphery in constricted areas, 9.3–14.2 × 8.4–12.5 μm (*x̄* = 12.7 × 10.8 μm, n = 40), somewhat obovoid, deeply constricted at the septa, flat from side view, frequently with attached conidiogenous cells when splitting from the conidiophores.

##### Culture characteristics.

Conidia germinating on PDA within 12–15 h, germ tubes produced from one or several cells. Colonies growing on PDA, reaching a diameter of 46 mm after 14 d at 25 °C, greyish white, unevenly raised, surface rough, moderately dense, radially striated at center, margin crenulate; reverse white to greyish white.

##### Material examined.

Thailand, Nan Province, on a dead leaf of *Musa* sp. (Musaceae), 12 September 2018, B.C. Samarakoon, BNS 069 (MFLU 19-2907, **holotype**), ex-type living culture MFLUCC 20-0001.

##### Notes.

Based on BLASTn search results of SSU, LSU, ITS and TEF1-α sequence data, *Spegazzinia musae* showed a high similarity (SSU = 98.24%, LSU = 98.92%, ITS = 96.91%, TEF1-α = 98.11%) to *S. neosundara* (MFLUCC 15-0456). In the multigene phylogeny, *S. musae* groups as a sister taxon to *S. deightonii* with strong statistical support (77% ML, 0.99 BYPP) (Figure 1). Also, ITS sequence comparison revealed 3.75% base pair differences between *S. musae* and *S. deightonii*, which is in agreement with the species concept outlined by Jeewon and Hyde (2016). Besides, *S. musae* has contrasting morphological features to *S. deightonii* in both kinds of conidia. The disk-shaped conidia of *S. musae* are 4-celled and do not bear spines at the periphery of cells, while the disc-shaped conidia of *S. deightonii* are 8-celled and spiny. Based on contrasting morphological differences and significant statistical support from our molecular phylogeny, *Spegazzinia musae* is introduced as a new species.

**Figure 3.**
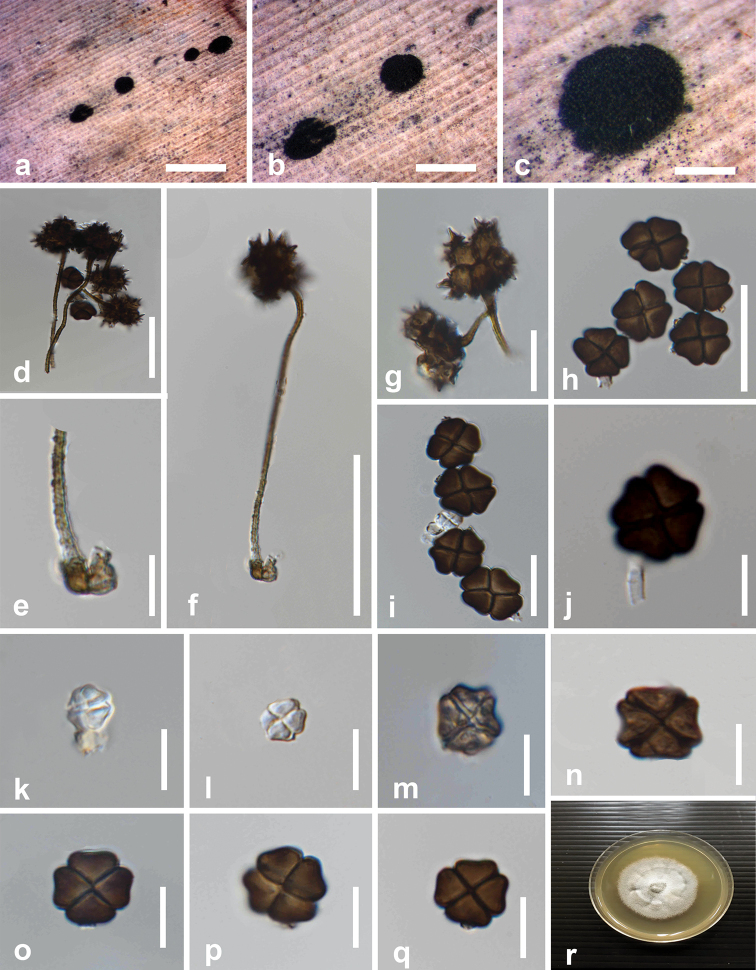
*Spegazzinia musae* (MFLU 19-2907, holotype) **a–c** fungal colonies on host surface **d** mature conidia **e** conidiophore of α conidia with the mother cell **f, g** α conidia **h–q** β conidia **r** colony on PDA after 28 days. Scale bars: 200 μm (**a–c**), 20 μm (**d–g, j**), 10 μm (**h, i, k–q**).

## Discussion

*Spegazzinia* is ubiquitous in the environment. Several taxa of *Spegazzinia* occur as saprobes on dead material of tropical, subtropical and temperate vascular plants (Ellis 1971; Subramanian 1988; Caretta et al. 1999; Delgado-Rodríguez et al. 2002; Bhat 2010; Leão-Ferreira and Gusmão 2010; Manoharachary and Kunwar 2010). In addition, *Spegazzinia* was also recorded from soil (Ellis 1971), dredged sediments of marine and brackish estuaries (Borut and Johnson 1962) and grassland vegetation (Caretta et al. 1999). *Spegazzinia tessarthra* was recorded as an endophyte from lichens (Manish et al. 2014) and recently *S. bromeliacearum* was introduced as an endophyte from the leaves of *Tilandsia catimbauensis* (Crous et al. 2019). Damon (1953) considered *S. tessarthra* to be an important decomposer of monocotyledonous plants and other cellulose containing materials in tropical and subtropical areas. *Spegazzinia deightonii* was previously recorded on monocotyledons such as *Areca catechu* (China, Taiwan; Matsushima 1980), *Cocos nucifera* (China; Tianyu et al. 2009) and *Panicum maximum* (Hong Kong; Lu et al. 2000) (Farr and Rossman 2020). Our study presents the first report of *Spegazzinia deightonii* in Musaceae as a saprobe and introduces our new species, *S. musae*.

There does not appear to be any host-specificity as the genus is found on a wide range of hosts in various habitats and there are no records of a pathogenic lifestyle. Some *Spegazzinia* species (such as *S. tessarthra*) have been identified as saprobes and endophytes and therefore the genus may have the potential of switching nutritional modes during the degradation of plant material (Promputtha et al. 2007).

*Spegazzinia* is a unique taxon among other dematiaceous hyphomycetes due to its conidial morphology and basauxic conidiogenesis. Most *Spegazzinia* species have contrasting morphological features in the shapes of α and β conidia. Some taxa bear spines in both types of conidia while some taxa do not bear spines. Simultaneously, some species of *Spegazzinia* such as *S. radermacherae*, *S. tessarthra* show similar characters in morphology apart from dimensions of conidia. The length of conidiophores can be varied with the environmental stresses (Cole 1974). Therefore, the use of morphological data coupled with DNA sequence data (SSU, LSU, ITS and TEF-α) will be crucial for better taxonomic resolutions in this genus.

*Dictyoarthrinium* (Apiosporaceae) bears some similar morphological features to *Spegazzinia* such as basauxic conidiogenesis (Ellis 1971) and cross septate, 4-celled, dematiaceous conidia with warts (Rao and Rao 1964). However, generic placement of *Dictyoarthrinium* in Apiosporaceae was confirmed by Vu et al. (2019) based on the LSU sequence of *D. sacchari* strain CBS 529.73. Therefore, *Dictyoarthrinium* was treated as a distinct genus with *Spegazzinia* (Vu et al. 2019).

Microfungal studies in *Musa* sp. are mostly oriented towards pathogens and endophytes due to the economic value of the fruit crop. Most of the pathogenic species descriptively studied from *Musa* sp. are identified as *Colletotrichum*, *Fusarium*, *Mycosphaerella*, *Neocordana* and *Phyllosticta* (Giatgong 1980; Wulandari et al. 2010; Churchill 2011; Guarnaccia et al. 2017; Marin-Felix et al. 2019; Maryani et al. 2019). The endophytic fungal populations of *Musa* sp. were studied by Brown et al. (1998), Photita et al. (2001a, 2004) and Samarakoon et al. (2019). Few studies have documented the saprobic diversity of *Musa* sp. and as we believe that there are saprobic niches associated with *Musa* sp. that are still unrevealed, taxonomists should investigate this hidden diversity for conservation purposes.

## Supplementary Material

XML Treatment for Spegazzinia deightonii

XML Treatment for Spegazzinia musae
